# Imaging Long-Term Fate of Intramyocardially Implanted Mesenchymal Stem Cells in a Porcine Myocardial Infarction Model

**DOI:** 10.1371/journal.pone.0022949

**Published:** 2011-09-01

**Authors:** Emerson C. Perin, Mei Tian, Frank C. Marini, Guilherme V. Silva, Yi Zheng, Fred Baimbridge, Xin Quan, Marlos R. Fernandes, Amir Gahremanpour, Daniel Young, Vincenzo Paolillo, Uday Mukhopadhyay, Agatha T. Borne, Rajesh Uthamanthil, David Brammer, James Jackson, William K. Decker, Amer M. Najjar, Michael W. Thomas, Andrei Volgin, Brian Rabinovich, Suren Soghomonyan, Hwan-Jeong Jeong, Jesse M. Rios, David Steiner, Simon Robinson, Osama Mawlawi, Tinsu Pan, Jason Stafford, Vikas Kundra, Chun Li, Mian M. Alauddin, James T. Willerson, Elizabeth Shpall, Juri G. Gelovani

**Affiliations:** 1 The Texas Heart Institute at St. Luke's Episcopal Hospital, Houston, Texas, United States of America; 2 Department of Experimental Diagnostic Imaging, The University of Texas M.D. Anderson Cancer Center, Houston, Texas, United States of America; 3 Department of Stem Cell Transplant and Cellular Therapy, The University of Texas M.D. Anderson Cancer Center, Houston, Texas, United States of America; 4 Department of Veterinary Surgery and Medicine, The University of Texas M.D. Anderson Cancer Center, Houston, Texas, United States of America; 5 Department of Nuclear Medicine, The University of Texas M.D. Anderson Cancer Center, Houston, Texas, United States of America; 6 Department of Imaging Physics, The University of Texas M.D. Anderson Cancer Center, Houston, Texas, United States of America; Stanford University Medical Center, United States of America

## Abstract

The long-term fate of stem cells after intramyocardial delivery is unknown. We used noninvasive, repetitive PET/CT imaging with [^18^F]FEAU to monitor the long-term (up to 5 months) spatial-temporal dynamics of MSCs retrovirally transduced with the sr39HSV1-tk gene (sr39HSV1-tk-MSC) and implanted intramyocardially in pigs with induced acute myocardial infarction. Repetitive [^18^F]FEAU PET/CT revealed a biphasic pattern of sr39HSV1-tk-MSC dynamics; cell proliferation peaked at 33–35 days after injection, in periinfarct regions and the major cardiac lymphatic vessels and lymph nodes. The sr39HSV1-tk-MSC–associated [^18^F]FEAU signals gradually decreased thereafter. Cardiac lymphography studies using PG-Gd-NIRF813 contrast for MRI and near-infrared fluorescence imaging showed rapid clearance of the contrast from the site of intramyocardial injection through the subepicardial lymphatic network into the lymphatic vessels and periaortic lymph nodes. Immunohistochemical analysis of cardiac tissue obtained at 35 and 150 days demonstrated several types of sr39HSV1-tk expressing cells, including fibro-myoblasts, lymphovascular cells, and microvascular and arterial endothelium. In summary, this study demonstrated the feasibility and sensitivity of [^18^F]FEAU PET/CT imaging for long-term, in-vivo monitoring (up to 5 months) of the fate of intramyocardially injected sr39HSV1-tk-MSC cells. Intramyocardially transplanted MSCs appear to integrate into the lymphatic endothelium and may help improve myocardial lymphatic system function after MI.

## Introduction

Stem cell therapy has shown promise in patients with acute myocardial infarction (MI) or chronic ischemia [1]. However, the mechanisms by which cells transplanted into the myocardium provide benefits are not well defined, in part, because the long-term fate of injected cells is unknown. Understanding the dynamics of cell trafficking and the long-term rates of cell survival and engraftment is essential for determining the mechanisms of cell therapy. Although clinically applicable imaging modalities are available for monitoring cardiac cell-based therapy [2]–[4], each has inherent disadvantages for studying the long-term fate of transplanted cells. Direct labeling of cells for single photon emission computed tomography (SPECT) and positron emission tomography (PET)/computed tomography (CT) is limited by the physical decay and biologic clearance of the radionuclide used [5], [6]. Paramagnetic contrast agents and supramagnetic nanoparticles for magnetic resonance imaging (MRI) not only have the disadvantage of biologic clearance, but they can also be sequestered in other cell types, limiting the duration of monitoring and increasing false positive results. A new approach—reporter gene imaging—has distinct advantages over direct labeling of cells. The reporter is expressed only in living cells and passes on to daughter cells during proliferation, so the rate of radiolabel decay or loss of nanoparticles from the labeled cells is not an issue. Moreover, this approach allows for repetitive imaging with PET or SPECT over months to years.

Herpes virus type 1 thymidine kinase (*HSV1-tk*) and its substrate-specific mutant derivatives *sr39HSV1-tk* [7] or *A168H-HSV1-tk* [8] have been widely used in reporter gene imaging [9]–[11]. The HSV1-TK reporter enzyme phosphorylates the radiolabeled nucleoside analogues (FHBG, FEAU, or FIAU) that are trapped inside the HSV1-tk transduced cell but not in nontransduced cells [12]–[15]. After intravenous administration of a radiotracer, the cells can be detected noninvasively by PET or PET/CT imaging. This reporter gene imaging approach with PET/CT has been used recently in clinical studies in human patients, including imaging of sr39HSV1-tk-based gene therapy of cancer [16] and track the localization of sr39HSV1-tk transduced T cells [17]. We [18], [19] and others [20] have shown the efficacy of PET imaging of HSV1-tk reporter gene–expressing stem cells in preclinical studies [18]–[20], including studies of short-term stem cell fate after intramyocardial injection in rodent models of MI [21]–[23], mesenchymal stem cells (MSCs) implanted in pig hearts [24], [25], and adenoviral-mediated HSV1-tk gene expression in pig hearts [26] demonstrating that this technology can be used in a large animal model. However, long-term (several months), repetitive imaging studies on the spatial and temporal dynamics of stem cells injected into the myocardium, their trafficking and engraftment, and persistence of stem cell–derived tissues have not been reported yet.

In this study, we have demonstrated the feasibility and sensitivity of [^18^F]FEAU PET/CT imaging for long-term *in vivo* monitoring (up to 5 months) of MSCs expressing the sr39HSV1-tk reporter gene after NOGA-guided transendocardial injection in a pig model of MI. We have assessed the biodistribution, survival, and long-term engraftment of transplanted cells and found that transplanted cells exhibit a biphasic distribution pattern in the heart, peaking in the infarct region at 4 to 5 weeks after delivery. Furthermore, transplanted cells appear to engraft as lymphovascular endothelial cells in myocardial lymphatic vessels and lymph nodes.

## Results

### Isolation, expansion, and characterization of pig MSCs and MSC-SR39TK

**Figure 1:**
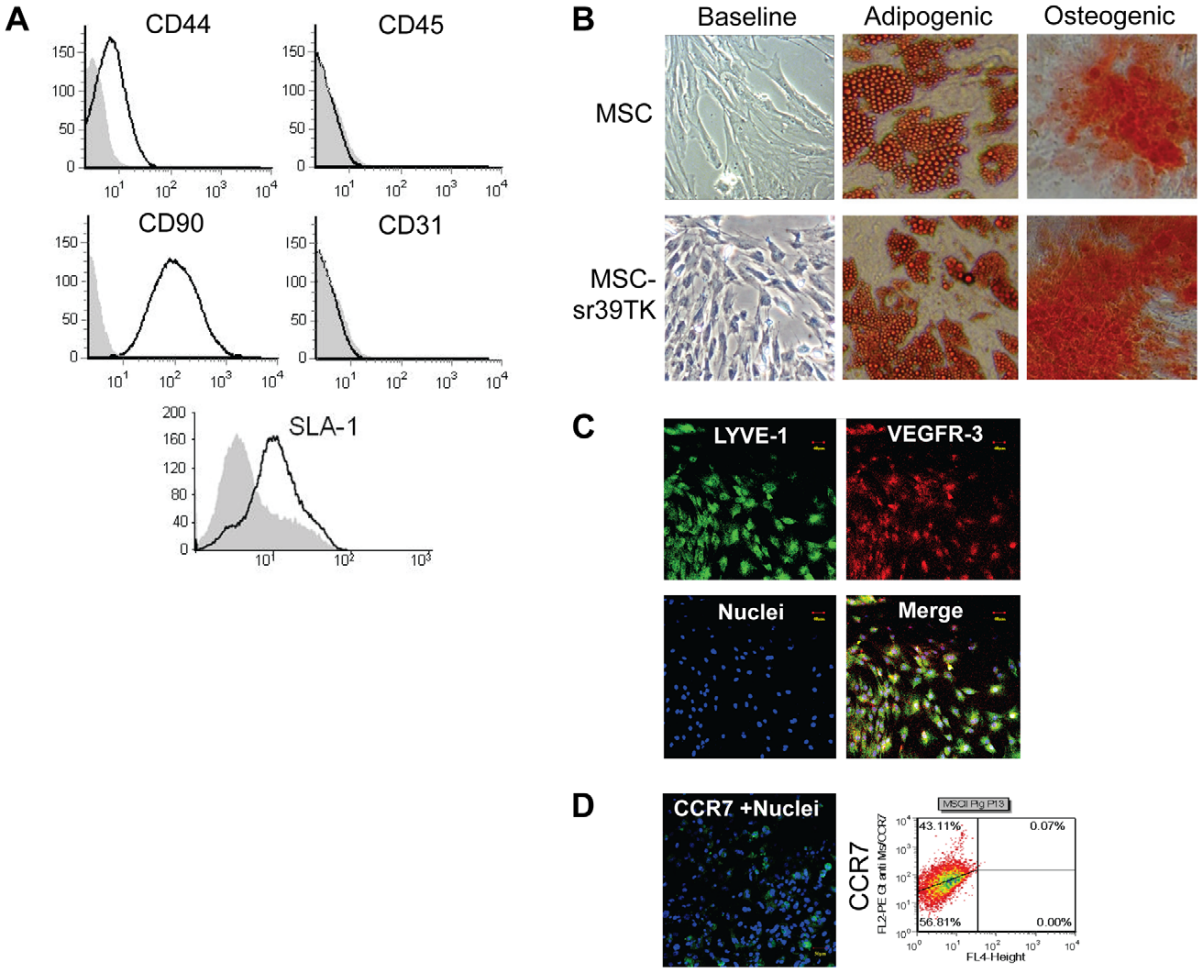
Characterization of porcine MSCs and sr39HSV1-tk-MSCs. (**A**) Expression of CD44, CD45, CD90, porcine SLA1, and CD31 in isolated MSCs, confirming the MSC nature of the cell population. (**B**) Morphology of porcine MSCs before and after sr39HSV1-tk reporter gene transduction is similar (baseline). These MSCs can be differentiated along the adipogenic lineage (oil-red O staining, left column) and the osteogenic lineage (alkaline phosphatase activity, right column). (**C**) The MSCs express LYVE-1 (green) and VEGFR-3 (red) in all cells, Hoeschst 33342-stained nuclei (blue), and (**D**) CCR7 (green) in approximately 43% cells (FACS).

After isolation, the bone marrow–derived pig MSCs were expanded *in vitro* for 2–3 weeks. The adherent, spindle-shaped MSCs expressed CD44, CD90, and the pig-specific SLA1 antigen, but were negative for the pan-hematopoietic marker CD45 and the endothelial marker CD31 (**Fig. 1A**). There were no significant morphologic differences in the MSCs before and after retroviral transduction with the sr39HSV1-tk reporter gene. MSCs from the 2nd passage were used to assess their capacity to differentiate into bone and fat cells. The MSCs differentiated along both the adipogenic pathway (oil red staining) and the osteogenic lineage (alkaline phosphatase activity) (**Fig. 1B**). The sr39HSV1tk-MSCs demonstrated similar differentiation abilities. In addition to CD90, the pig MSCs expressed the lymphoendothelial markers LYVE-1 and VEGFR-3 (**Fig. 1C**), enabling them to respond to lymphangiogenic stimuli. Approximately 43% cells expressed CCR7 (**Fig. 1D**), enabling them to respond to inflammatory stimuli.

### In vivo imaging for sr39HSV1-tk-MSC tracking, engraftment, and retention

No procedural complications occurred in any pig during any procedure, including death, cardiac perforation, ventricular fibrillation, or tachycardia. Group I pigs (negative control; n = 3) were injected with nontransduced autologous MSCs. No specific accumulation of [^18^F]FEAU was observed anywhere in the heart (average standardized uptake value [SUV], 0.32±0.04) or in any other tissues, except for the kidneys and bladder, which are involved in the excretion of [^18^F]FEAU from the body. Overall, the biodistribution of [^18^F]FEAU in normal pigs appeared to be similar to that observed by us in rhesus macaques [27].

**Figure 2:**
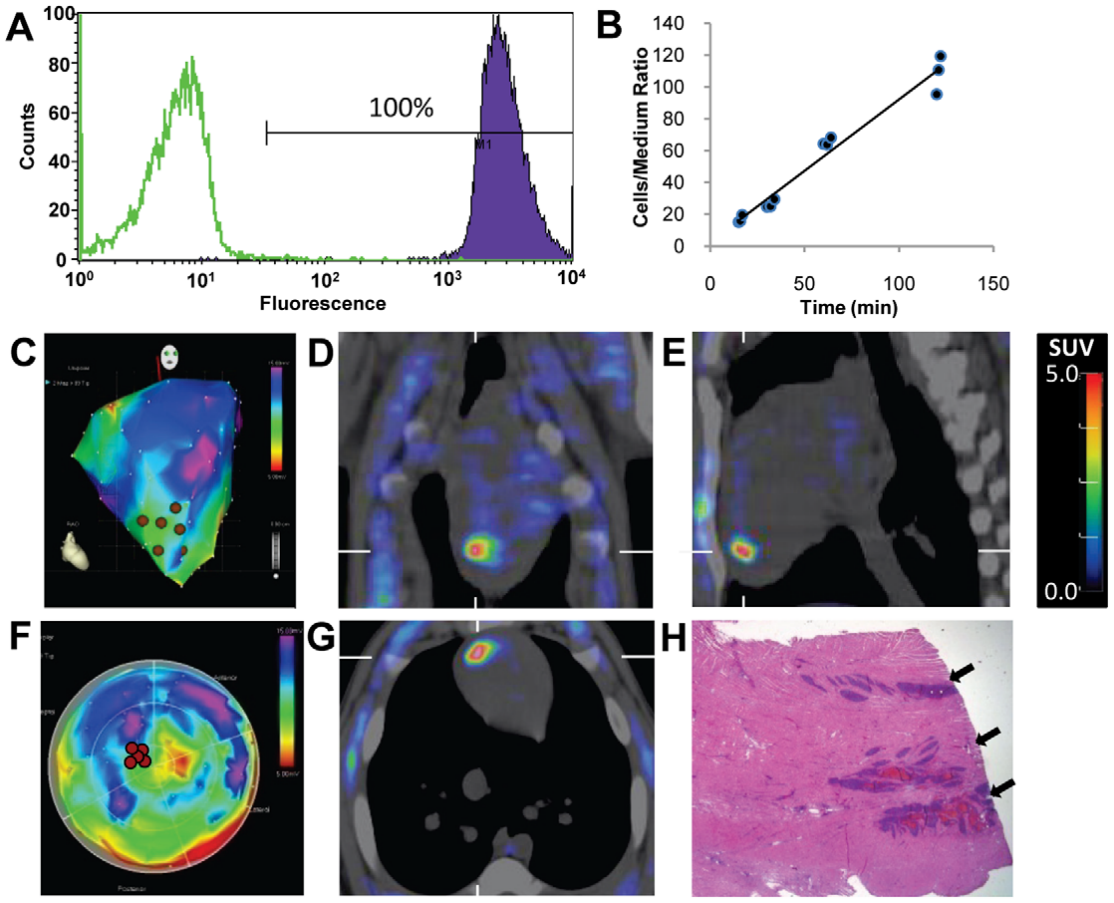
*In vitro* characterization and *in vivo* [^18^F]FEAU PET/CT of sr39HSV1tk-Wi-38 cells. (**A**) The level of LNGFR and HSV1-TK co-expression in sr39HSV1tk-Wi-38 (purple) non-transduced Wi-86 fibroblasts (green). (**B**) Accumulation of [^3^H]FEAU over time in sr39HSV1tk-Wi-38 cells *in vitro* (influx rate Ki = 0.90±0.05 ml/g/min) reflects a very high level of *sr39HSV1-tk* reporter gene expression in this cell population. (**C,F**) NOGA maps indicating sites of injection of sr39HSV1tk-Wi-38 cells (brown dots). (**D**) Coronal, (**E**) sagittal, and (**G**) axial PET/CT images of the pig thorax obtained 1 hour after [^18^F]FEAU administration and 6 hours after intramyocardial injection of sr39HSV1tk-Wi-38 cells clearly demonstrate the localization of cells injected into the myocardium. (**H**) H&E stained section of myocardium showing deposits of sr39HSV1tk-Wi-38 cells in the area of injection (arrows).

In Group II pigs (positive control; n = 2), sr39HSV1tk-Wi-38 cells (Wi-38 murine fibroblasts transduced with *sr39HSV1-tk* reporter gene) were used for intramyocardial injections. The level of sr39HSV1-tk expression in the sr39HSV1tk-Wi-38 cell line was very high, as inferred by the level of LNGFR coexpression (**Fig. 2A**) and the rapid [^3^H]FEAU accumulation observed in *in vitro* studies (influx rate *Ki* = 0.90±0.05 ml/g/min; **Fig. 2B**). Consequently, high levels of [^18^F]FEAU accumulation (SUV, 2.4±0.3) were observed in the area of cell injections into the left ventricular apical septum (**Fig. 2C–G**). To confirm that the sr39HSV1tk-Wi-38 cells were responsible for the increased FEAU signal detected in the myocardium, the pigs were euthanized 1 day after the [^18^F]FEAU PET/CT imaging study to obtain tissues for histopathologic analyses of the injection sites. Sr39HSV1tk-Wi-38 cell deposits were clearly identified in the myocardial tissue at the sites of injection (**Fig. 2H**), confirming the source of strong signals in [^18^F]FEAU PET/CT images.

**Figure 3:**
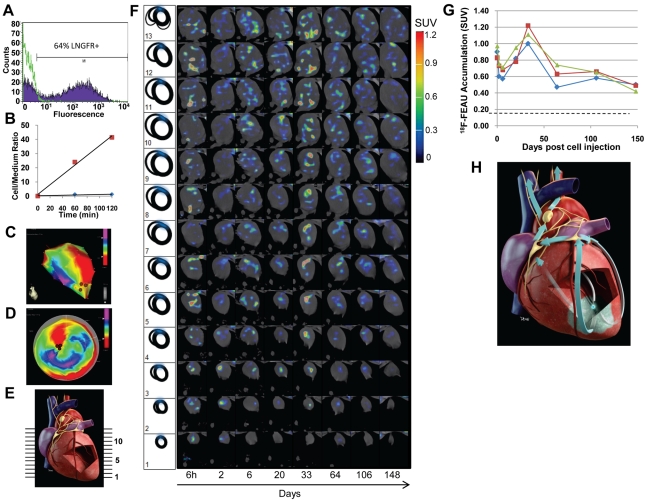
*In vitro* characterization and repetitive long-term imaging of intramyocardially injected sr39HSV1-tk-MSC cells with [^18^F]FEAU PET/CT. (**A**) The level of LNGFR co-expression with sr39HSV1-TK in transduced sr39HSV1-tk-MSC (purple) and non-transduced porcine MSCs (green). (**B**) [^3^H]FEAU accumulation was moderate in sr39HSV1-tk-MSC (red squares) (Ki = 0.34±0.03 ml/g/min) but negligible in naïve pig MSCs (blue diamonds). (**C,D**) NOGA maps of sr39HSV1-tk-MSC injection sites into the myocardium. (**E**) Cross-sectional model of the heart indicating the image planes numbered on the right. (**F**) [^18^F]FEAU PET/CT images of the pig heart at different time points after intramyocardial transplantation of sr39HSV1-tk-MSCs. A schematic representation of the ventricles (thick ovals), the atria (thin ovals), and the infarct area (blue) is provided in the left column. (**G**) Dynamics of regional [^18^F]FEAU accumulation in the injection site (slice 5, blue diamonds), the anterior septum-left ventricular wall region of infarct (slice 6, red squares), and the aortic lymph node (slice 12, green triangles). (**H**) Artist's representation of the observed patterns of sr39HSV1-tk-MSC distribution from the injection site to the infarct area (blue) and beyond through the myocardial muscle and lymphatic system (arrows).

In **Group III** pigs (test group; n = 3), 10^8^ sr39HSV1tk-MSCs (autologous porcine MSCs transduced with *sr39HSV1-tk* reporter gene) were used for intramyocardial injections. The sr39HSV1tk-MSCs exhibited moderate levels of *sr39HSV1-tk* expression as measured by coexpression of LNGFR (52–64% cells; **Fig. 3A**) and *in vitro* accumulation of [^3^H]FEAU (influx rate *Ki* = 0.34±0.05 ml/g/min; **Fig. 3B**). In 2 pigs, [^18^F]FEAU PET/CT imaging studies were performed at 4 hours, and 7, 21, and 35 days after sr39HSV1-tk-MSC injection (**Figs. S1 and S2**); the 2 pigs were euthanized for histopathologic analyses of cardiac and extracardiac tissues on day 35 after the last imaging. The third pig was imaged with [^18^F]FEAU PET/CT at 6 hours and 2, 6, 20, 33, 64, 106, and 148 days after MSC injection into the left ventricular apical septum (**Fig. 3C,D**) and then euthanized on day 150.

The vascular anatomy and anatomic orientation of the heart inside the thorax differ in humans and pigs [28], [29]. Because the pig heart hangs more vertically within the thorax, the axial images of the apex (**Fig. 3E,F**; slices 1–4) are formed almost entirely by the left ventricle. Therefore, to simplify the description of PET/CT imaging results in **Figure 3**, we have provided a schematic diagram showing the anatomic relationship between the left and right ventricles (thick ovals) and the atria (thin ovals) (**Fig. 3F** **;** first column) for each of the image planes shown on the cross-sectional model of the heart (**Fig. 3E**). The corresponding 12-hour clock coordinates were used to demarcate the localization of the imaging signals.

The results of repetitive PET/CT imaging of the pig monitored for 148 days after injection of sr39HSV1-TK-MSC are presented in detail in **Figure 3**; the results obtained in the other 2 pigs monitored for 35 days are presented in the supplementary materials (**Figs. S1 and S2**). The initial site of sr39HSV1-tk-MSC injection into the apical septum of the left ventricle (**Fig. 3C,D**) was clearly identifiable in all animals on [^18^F]FEAU PET/CT images obtained at 6 hours after sr39HSV1-tk-MSC injection (**Fig. 3F**, 6 h column, slices 1–5, signal in the center). At 6 hours after intramyocardial injection, the sr39HSV1tk-MSCs were already clearly detectable in the area of infarct (**Fig. 3F**, 6 h column, slices 4–8, at 11 to 1 o'clock) and in the area of the posterior interventricular sulcus and vena cava (6 h column, slices 8–12, at 7 o'clock). At 2–6 days after sr39HSV1-tk-MSC injection, the [^18^F]FEAU signal rapidly decreased in all areas of the heart (**Fig. 3F**, 2 and 6 day columns, slices 1–8). However, from days 6 to 33 after injection, the [^18^F]FEAU signal gradually increased both at the initial site of injection (**Fig. 3F**, 6, 20, and 33-day columns, slices 1–5, central area) and at the anterior portion of the septum adjacent to the periinfarct area (**Fig. 3F**, 6, 20, and 33-day column, slices 6–8, diagonally from the center to 1 o'clock); this effect was noted in all animals in this group. Two months after sr39HSV1-tk-MSC injection (**Fig. 3F**, 64-day column), the intensity of the [^18^F]FEAU signal decreased again dramatically in many areas of the heart, but was still clearly detectable at the injection site (**Fig. 3F**, 64-day column, slices 1–5, center), the anterior part of the septum adjacent to the infarct area (slices 6–10, diagonally from the center), in the area of the infarct (**Fig. 3F**, 64-day column, slices 6–8, 1 o'clock), the posterior interventricular lymphatic sinus (**Fig. 3F**, all days, slice 12, 7 o'clock), and the periaortic lymph nodes (**Fig. 3F**, 33 and 64-day column, slice 12 and 13 at 6 o'clock) The [^18^F]FEAU activity in these areas was stable or had minimally decreased during the subsequent 4 months (**Fig. 3F**, days 106 and 148). The dynamics of [^18^F]FEAU accumulation in the sr39HSV1-tk-MSC injection sites was quantified (**Fig. 3G** **)**; [^18^F]FEAU SUV was measured in the anterior septum, the left ventricular wall region of the infarct, and in the area of the posterior interventricular lymphatic sinus (**Fig. 3F**, all days, slice 12, 7 o'clock). **Figure 3H** shows a representation of the observed pattern of sr39HSV1-tk-MSC distribution from the injection site to the periinfarct region and to the base of the heart.

**Figure 4:**
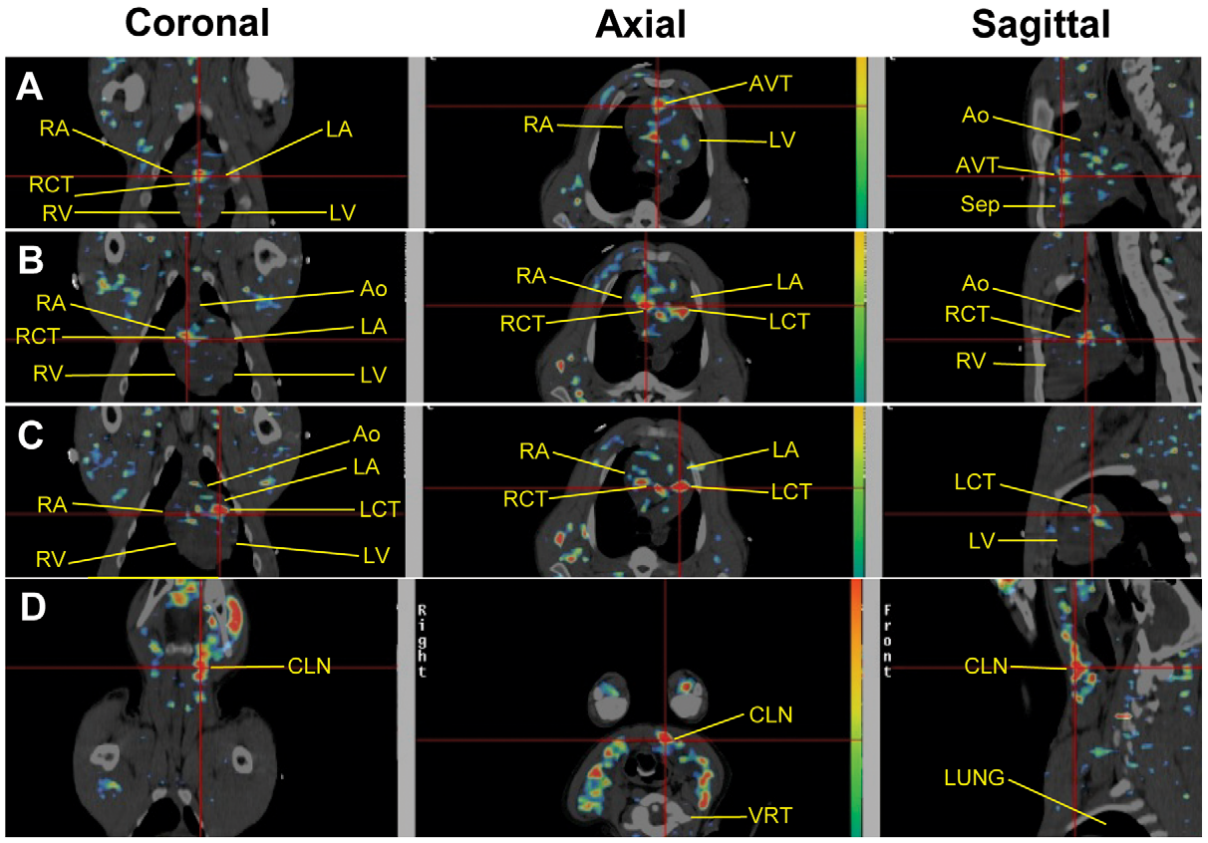
[^18^F]FEAU PET/CT images observed in a pig 35 days after intramyocardial injection of sr39HSV1tk-MSCs. PET/CT images of [^18^F]FEAU accumulation in the anterior interventricular lymphatic trunk, AVT (**A**), the right (**B**) and left (**C**) coronary lymphatic trunks (RCT and LCT, respectively), and the left cervical lymph nodes, CLN (**D**). The location of aorta (Ao), left atrium (LA), right atrium (RA), left ventricle (LV), and right ventricle (RV), interventricular septum (SEP), and cervical vertebral body (VRT) are annotated on the images.

In all 3 pigs in **Group III**, at different days after intramyocardial injection of sr39HSV1tk-MSCs, the [^18^F]FEAU activity was also observed in the periaortic lymphatic structures at the base of the heart and in the left and right coronary trunks (**Fig. 4A–C**), a finding that is consistent with the anatomy of the pig cardiac lymphatic system [30], [31]. In 2 pigs, the [^18^F]FEAU activity was also observed in the cervical lymph nodes, indicating the presence of sr39HSV1tk-exprerssing cells (**Fig. 4D** **;** **Fig. S3**).

### Histologic and immunohistochemical studies

**Figure 5:**
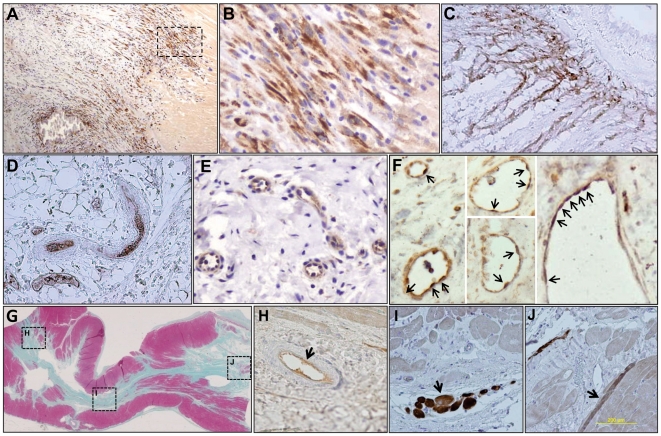
Tissue analysis. Histopathological and immunohistochemical analysis of tissue samples obtained from the sites of [^18^F]FEAU accumulation in pigs at 35 days (**A–F**) and 150 days (**G–I**) after intramyocardial injection of sr39HSV1tk-MSCs. (**A**) The sr39HSV1-TK positive cells are densely integrated within the interface of scar and normal-appearing myocardial tissue (magnification ×100); (**B**) Higher magnification (×400) of the area in panel **A** identified by a black dotted line. The sr39HSV1-TK positive cells are observed in (**C**) the vascular-type structures inside the scar tissue, (**D**) the anterior interventricular lymphatic vasculature, and (**E**) peri-aortic lymph node. Expression of lymphovascular-specific marker LYVE-1 (brown) in the myocardial scar tissue counterstained with hematoxylin (blue). (**F**) Dual immunohistochemical staining for LYVE-1 (brown) and sr39HSV1-TK (dark blue) demonstrated sr39HSV1tk-MSCs-derived lymphovascular endothelial cells (arrows) in small and medium-size lymphatic vessels in the myocardial scar tissue and large pericardial lymphatic vessels next to the scar tissue. (**G**) Gross images of H&E stained sections of cardiac wall segments containing post-infarct scar and adjacent myocardial tissues (at 150 days); dotted lines identify areas shown in panels H–J. Immunohistochemical staining for sr39HSV1-TK expression (brown) is observed in arterial endothelial cells (**H**), fibromyoblast-like cells (**I**), and, occasionally, as striated myofibers (**J**).

Histopathologic and immunohistochemical (IHC) analyses of myocardial tissue sections obtained at 35 days after injection of sr39HSV1tk-MSCs demonstrated large numbers of sr39HSV1-tk-MSC–derived cells at the interface of scar and the myocardial “penumbra” (**Fig. 5A,B**), consistent with the increased [^18^F]FEAU signal in the periinfarct regions observed with PET/CT 33–35 days after cell injection. The sr39HSV1-TK positive cells were present in the myocardial lymphatic vessels and the peri-aortic lymph node (**Fig. 5C,D**), which was also consistent with [^18^F]FEAU PET/CT imaging results (**Fig. S4**), and in the cervical lymph nodes (**Fig. S3**). The %HSV1-tk+ cells ranged from 2% in the periphery of the periinfarct zone to 79±12% in the areas adjacent to the developing scar (i.e., Fig. 5A,B); lymphatic microvessels within the area of the scar had segments developed from almost 100% HSV1-tk+ cells (as seen in Fig. 5C, E, and F); and the lymph nodes contained 64±8% HSV1-tk+ cells (Fig. 5D). IHC staining for the lymphovascular-specific marker LYVE-1 revealed an extensive network of lymphatic microvessels inside the developing scar tissue (**Fig. 5E**). Dual IHC staining for LYVE-1 and sr39HSV1-TK demonstrated multiple dual-labeled lymphovascular endothelial cells in the infarct region and the remote areas of myocardium (**Fig. 5F**). Dual IHC staining of myocardial tissue sections with antibodies for anti-HSV1-TK and anti-αSMA demonstrated the coexpression of these proteins in sr39HSV1-tk-MSC–derived fibromyoblasts (**Fig. S4**). These findings demonstrate that sr39HSV1tk-MSCs integrated into the developing fibrovascular tissue and lymphatic vasculature.

IHC analysis of myocardial tissue samples obtained 150 days after the sr39HSV1-tk-MSC injection confirmed the presence of sr39HSV1-tk-MSC–derived cells in the regions corresponding to sites of [^18^F]FEAU accumulation in PET/CT images. Sr39HSV1-tk-MSC–derived cells were observed within fibrous connective tissue of the postinfarct scar, as well as dispersed within microvascular, lymphovascular, and arterial endothelial cells (**Fig. 5G–J**).

### Cardiac MRI lymphography

**Figure 6:**
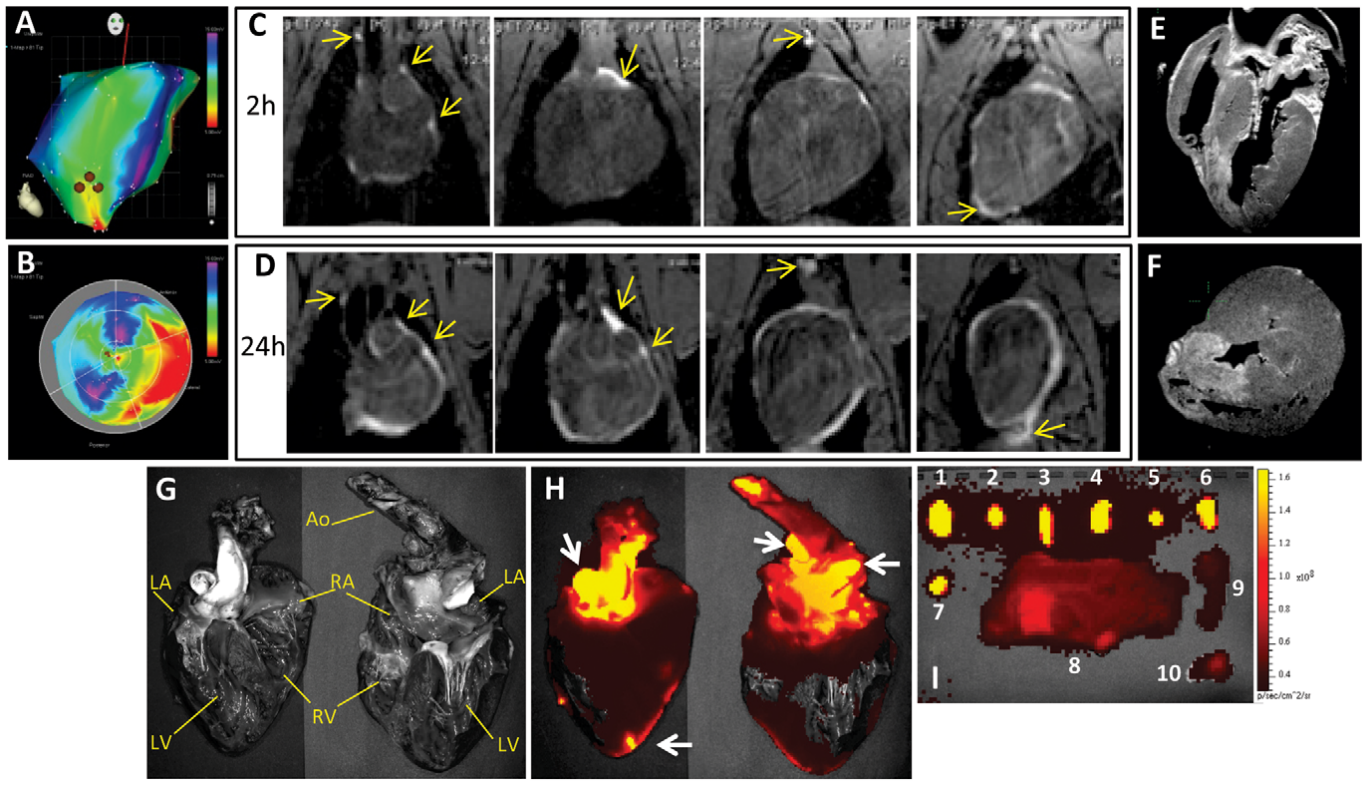
MR and near infrared fluorescence lymphography with PG-Gd-NIRF813. (**A, B**) NOGA maps indicate the injection of PG-Gd-NIRF813 contrast agent in the area of anterior interventricular septum. MR-lymphagraphy images (at 1.5T) were obtained at 2 hours (**C**) and 24 hours (**D**) post intramyocardial injection of PG-Gd-NIRF813. Increased T1 signal was observed in the pericardium, major cardiac lymphatic vessels, periaortal and mediastinal lymph nodes, as well as in the diaphragmal adhesion (arrows). (**E, F**) *Ex vivo* MR imaging (at 4.7T) of the same heart post excision demonstrated increased signal within the septum corresponding to the location of the myocardial infarction (**E** – coronal image; **F** – sagittal image). (**G**) Macro photograph of the excised and coronally sectioned heart: LA – left atrium; LV – left ventricle; Ao – aorta; RA – right atrium; RV – right ventricle. (**H**) Near infrared fluorescence images demonstrate the correspondence of a bright signal to the injection site and different lymphatic structures of the heart (arrows). (**I**) Excised lymph nodes on NIRF images were intensely fluorescent: 1 and 3 - paratracheal; 2 - subaortic; 3 - paratracheal; 4 - bronchopulmonary; 5 – superior phrenic; 6 - dorsal; 7 - carinal lymph node; 8 - cervical tissue; 9 and 10 - cervical lymph nodes.

The pathways of lymphatic outflow from the site of sr39HSV1-tk-MSC injection were mapped in the one pig in **Group IV**. In this pig, after creation of the infarct, the PG-Gd-NIRF813 contrast agent was injected into the left ventricular apical septum by using NOGA electromechanical mapping in a manner similar to the injection of sr39HSV1tk-MSCs (**Fig. 6A,B**). Two hours after injection of PG-Gd-NIRF813, the pig underwent *in vivo* MR imaging. T1-weighted MRI (1.5T) revealed contrast enhancement of the injection site and the epicardium-pericardium; contrast enhancement was especially prominent at the anterior interventricular sulcus, the periaortic lymph nodes, and the area at the base of the heart (**Fig. 6C**). These findings are consistent with the anatomy of the lymphatic system of a porcine heart. At 24 hours after contrast injection, MR images of the same pig demonstrated increased intensity of contrast enhancement of the epicardium-pericardium of the whole heart, the periaortic lymph nodes (**Fig. 6D**), and the infarcted region. The pig was euthanized 28 hours after contrast injection, and high resolution *ex vivo* MRI (4.7T) of the excised heart was performed to validate the results of *in vivo* MR imaging (**Fig. 6E,F**). *Ex vivo* MRI confirmed the distribution of the contrast agent from the site of injection into the infarcted region and through the subepicardial network of lymphatic microvessels into the anterior and posterior interventricular lymphatic trunks, the left and right coronary lymphatic trunks, the main coronary lymphatic trunk, and the periaortic lymph nodes (**Figs. S5, S6**).

### Near-infrared fluorescence imaging

Near-infrared fluorescence imaging of the dissected heart (in the one pig from Group IV) confirmed the pattern of distribution of contrast agent seen with *ex vivo* and *in vivo* MRI (**Fig. 6G–I**). The contrast agent was distributed at the site of injection, the myocardial lymphatic system, and the periaortic lymph nodes.

### Echocardiography

Cardiac contractile function was measured by echocardiography in 2 pigs from the test group (Group III) after sr39HSV1-tk-MSC injection (**Tab. 1**). There were no substantial differences in any parameter between the 2 pigs on injection day. The LVEF improved by 19% on Day 28 in one pig (#8s210) and by 12% on Days 21 and 28 in the other pig (#8s211).

**Table 1:** Echocardiography results.

Pig ID #	Days post injection	IVS(cm)	PW(cm)	LVEDD(cm)	LVESD(cm)	LVEF(%)
#8s210	0	0.8	0.7	3.7	2.7	53
	7	0.9	0.8	4.8	3.6	50
	21	0.8	0.7	4.4	3.1	57
	28	0.6	0.7	4.6	3.1	63
	35	0.6	0.7	5	3.7	54

Pig ID #	Days post injection	IVS(cm)	PW(cm)	LVEDD(cm)	LVESD(cm)	LVEF(%)
#8s211	0	1.1	0.6	3.9	2.7	59
	7	0.8	0.8	5.1	3.2	67
	21	1	0.7	4.9	3.2	66
	28	1	0.7	4.7	3.2	66
	35	1	0.7	4.9	3.3	60

IVS, interventricular septum; LV, left ventricular; PW, posterior wall of left ventricular; LVEDD, left ventricular end-diastolic dimension; LVESD, LV end-systolic diameter; LVEF, left ventricular ejection fraction.

## Discussion

In the current study, we have demonstrated for the first time the feasibility of repetitive noninvasive PET/CT imaging with [^18^F]FEAU for long-term monitoring (up to 5 months) of intramyocardially injected autologous MSCs in a pig model of acute MI. Our imaging studies showed a biphasic biodistribution of transplanted cells, with cell proliferation peaking about 34 days after delivery. Furthermore, our findings indicate that transplanted MSCs may integrate as lymphatic endothelium, suggesting that cell therapy with MSCs may provide benefits by facilitating improvements in lymphatic outflow from the infarct area. This study lays the groundwork for the clinical translation of reporter gene technology, which overcomes the limitations associated with current techniques for monitoring the in-vivo fate of transplanted cells.

To date, only two groups have reported on successful imaging of intramyocardially administered MSCs expressing sr39HSV1-tk in pigs using PET/CT with [^18^F]FHBG [24], [25]. In a xenotransplantation study, Willmann and colleagues [25] assessed the efficacy of PET/CT imaging with [^18^F]FHBG of adenovirally transfected human MSCs performed once at 8 hours after open-chest surgery with direct intramyocardial injection in normal, not infarcted pigs. The latter xenotransplantation study demonstrated negative [^18^F]FHBG PET imaging results when the MSCs were injected in PBS, presumably due to a substantial loss of cells from the injection site. However, when the MSCs were injected in matrigel, their localization was confined to the site of injection and could be clearly visualized by [^18^F]FHBG PET.

In contrast, the current study in a porcine MI model demonstrated that retrovirally transduced sr39HSV1-tk-MSCs suspended in PBS and injected transendomyocardially into the periinfarct area using NOGA guidance, were clearly detectable in the injection site 4–6 hours later by [^18^F]FEAU PET/CT in all animals. Our results are in agreement with the report by Gyongyosi, et al. (2008) [24], which demonstrated that autologous porcine MSCs lentivirally transduced with truncated *HSV1-tk* in fusion with *Renilla luciferase* and *RFP* genes were detectable by [^18^F]FHBG PET at 8 hours post NOGA-mediated transendocardial injection into the left ventricular septum. One explanation for differences in results with Willmann et al, (2009) [25] may be that autologous pig MSCs have a higher affinity for the inflammatory and tissue repair milieu in the infarcted pig myocardium as compared to human MSCs xenotransplanted into the normal (uninfarcted) pig heart.

In the current study, [^18^F]FEAU accumulation at the site of MSC-tk injection decreased significantly (about 50%) over the first week after injection in 2 of the 3 animals but was still detectable by PET/CT. Gyongyosi and colleagues [24] reported detecting a faint signal in the heart by [^18^F]FHBG PET 7 days after injecting a significantly lower number of cells into two sites into the left ventricular septum, as compared to the number of cells injected intramyocardially in our study. Other studies in a porcine model of MI have demonstrated that only 3% to 11% of intramyocardially injected stem cells are retained at the injection site or in infarct areas a few days after delivery [3], [32]. In one study [3], after intramyocardial injection (open-chest surgery) of [^111^In]oxine-labeled autologous peripheral blood mononuclear cells in a porcine model of MI, only about 11% of cells were retained in the injection site 1 hour later; most radiolabeled cells were recovered in the lungs [3]. In our experience using the same catheter and mapping system in normal pigs as that used in the current study (unpublished data), we have recovered an average of 25% (range, 13–41%) of injected cells at the injection site 1 hour after delivery, as quantified by labeling cells with BioPal - a europium-containing, nontoxic colloidal lanthanide (BioPhysics Assay Laboratory, Inc.) that allows for quantitative measurement of retained cells on the basis of neutron activation of the europium atoms. The initial rapid decrease of MSC-tk–associated radioactivity observed in the present study is probably due to multiple factors. After injection, cell loss occurs mostly by venous drainage to the lungs, as described above [33]; or by subsequent cell death; backwash from the needle track may account for a small amount of cell loss as well [3]. Nevertheless, despite the cell loss observed during the first week after injection, the sr39HSV1tk-MSCs (and progeny) were still detectable by [^18^F]FEAU PET/CT in the site of injection in all 3 animals at 3 and 5 weeks after injection of the cells, and in one animal after 2, 3, and 5 months.

Importantly, our studies demonstrated that a large portion of sr39HSV1tk-MSCs had migrated into the infarcted area within 4 to 6 hours after intramyocardial injection, and some sr39HSV1tk-MSCs had migrated through the cardiac lymphatic vessels into the periaortic lymph nodes and even into the remote cervical lymph nodes. This trafficking of cells may relate to the pathophysiologic role of lymph flow in MI. The flow rate of interstitial lymphatic fluid inside the normal myocardium is at least 10 times greater than that in other tissues, such as lung or skeletal muscle [34]. The major determinant of myocardial lymph propulsion is the rhythmic contraction and relaxation of the heart [35]–[37]. During MI, the myocardial lymph flow is increased more than 50% but cannot compensate for the formation of myocardial edema [38]. Thus, the rhythmic contractions of the heart may push the injected MSCs along the pathways of increased lymphatic flow from the infarcted tissue into the anterior and posterior interventricular lymphatic trunks and into the periaortic cardiac lymph nodes. Moreover, our studies using MRI and near infra-red flourescence (NIRF) cardiac lymphography after intramyocardial injection of PG-Gd-NIRF813 suggest that MSCs may rapidly migrate because of hydrodynamic drag by increased edematous flow through the enlarged interstitial spaces and via the enlarged lymphatic channels and vessels, which are hallmarks of MI [38], [39].

We, for the first time, provide evidence that transplanted MSCs delivered directly into the heart after MI can engraft into the newly developing lymphatic vasculature within the periinfarct regions and the periaortic nodes. Specifically, histopathologic and immunohistochemical studies showed large numbers of sr39HSV1-TK and LYVE-1 positive cells engrafted into the lymphatic vasculature of the heart, the periaortic lymph nodes, and in some cases in remote cervical lymph nodes at 35 and 150 days after injection. These findings corroborate the results of *in vivo* [^18^F]FEAU PET/CT imaging studies. This distribution of transplanted cells may contribute to reconstitution of the function of the myocardial lymphatic system. The cardiac lymphatic system shows decreased function after MI that normalizes after restoration of perfusion [40]. Newly formed lymphatic vessels contribute to fibrosis and scar formation by draining proteins and edematous fluid [39]. At the time we injected sr39HSV1tk-MSCs (1 week after infarction), cardiomyocytes around the newly forming scar are known to express high levels of vascular endothelial growth factor (VEGF)-C [39], which may facilitate the differentiation of sr39HSV1tk-MSCs into lymphatic endothelium. At this point in the healing process, lymphatic drainage begins to improve in an attempt to resorb the flow of edematous fluid. Our hypothesis that transplanted MSCs participate in the lymphatic aspects of cardiac healing is supported by the results of current in vitro studies demonstrating that pig MSCs express lymphoendothelial markers LYVE-1, VEGFR-3, and CD-90, which enable the cells to respond to lymphoproliferative stimuli, as well as CCR7, a receptor that is crucial for recruitment of lymphatic endothelial precursors into sites of inflammation and injury [41]. Conrad, et al (2009) have also demonstrated that MSCs express CCR7, can acquire a lymphoendothelial phenotype when exposed to VEGF-C, and can then increase *in vivo* regeneration of the lymphatics in a mouse tail model of lymphedema [42]. Furthermore, our hypothesis is supported by the data indicating that infusion of hyaluronidase, a well-recognized lymphagogue, reduces myocardial infarction size by increasing the myocardial lymph flow rate [34], [43]–[45]. Because the lymphatic system of the pig heart is similar to that of the human heart [34], our results may have implications for understanding the therapeutic effect of intramyocardially administered MSCs in patients with MI.

After an initial decrease during the first week after injection, the [^18^F]FEAU signal in our study gradually increased. The peak signal was observed at the site of injection, in the infarcted myocardium, and in the major cardiac lymphatic vessels and the periaortic lymph nodes at 30 to 35 days after injection. This demonstrates that between 2 and 5 weeks after intramyocardial injection, the surviving MSCs proliferated at the infarct site and other local areas, yielding stronger signals from the progeny tissue observed at 33–35 days. Such a pattern of signal intensity is consistent with a general increased proliferative activity of various cell types in the myocardium (ie, fibroblasts, neovascular endothelial cells) during the 3 to 5 weeks after MI [46]. Also, our findings are in agreement with the report by Quevendo, et al (2009), in which viable MSCs were identified inside the infarct and border zones at 12 weeks post transplantation into the chronic ischemic scar [47]. The presence of large numbers of sr39HSV1-tk positive cells in the sites of high [^18^F]FEAU accumulation was confirmed by immunohistochemical studies of myocardial and remote (ie, lymph node) tissues in 2 of 3 pigs euthanized on day 35 after sr39HSV1tk-MSCs injection. In one pig, long-term repetitive [^18^F]FEAU PET/CT studies conducted after 1 month showed a gradual reduction of [^18^F]FEAU activity in the sites of sr39HSV1tk-MSCs engraftment at 64, 106, and 148 days after cell injection. This finding suggests a gradual decrease in the overall inflammation in the infarct and in the proliferation of different cell types during the later phases of fibrosis and myocardial scar maturation [46]. Other reasons for the observed decrease in signal over time may also reflect immunogenic processes as well as decreasing protein expression. Nevertheless, [^18^F]FEAU PET/CT imaging demonstrated sufficient sensitivity to detect the residual sr39HSV1-tk–expressing progeny tissues developed from the transplanted sr39HSV1tk-MSCs. The presence of sr39HSV1-tk positive cells in the sites of high [^18^F]FEAU accumulation was confirmed by immunohistochemical studies of myocardial tissue sections obtained 150 days after injection.

The long-term persistence of sr39HSV1tk-MSCs–derived progeny may have significance in terms of the therapeutic mechanism of intramyocardially injected MSCs. In preclinical studies, MSCs appear to provide benefits through a paracrine mechanism. MSCs have been shown to attenuate cardiac fibrosis by producing HGF [48] and to contribute to angiogenesis by producing VEGF [49]. Data from our group [50] suggest that MSC therapy in dogs may increase collagen deposition after MI, thus fortifying the nascent myocardial scar; MSC-treated dogs showed less unresolved infarct, with smaller areas of necrosis surrounded by collagen deposits. Improved healing associated with cell therapy may involve increased lymphatic function to help eliminate excess fluids and cells.

In summary, we have demonstrated the feasibility and sensitivity of [^18^F]FEAU PET/CT imaging for long-term monitoring (up to 5 months) of the *in vivo* fate of MSCs that express the sr39HSV1-tk reporter gene after NOGA-guided transendomyocardial injection in a pig model of Ml. Our *in vivo* imaging results were confirmed by immunohistochemical studies *in situ*. We found that transplanted MSCs have a biphasic pattern of spatial and temporal dynamics, with a peak migration (and/or proliferation) into the infarcted area at 4 to 5 weeks after intramyocardial injection in to the peri-infarct zone. We have discovered that MSCs incorporate into the cardiolymphatic system and into areas of myocardial healing, which has not been previously reported. We hypothesize that transplanted MSCs may contribute to the process of myocardial repair by engrafting into lymphatic vessels within the periinfarct regions and the periaortic nodes, thus facilitating improved lymphatic outflow from the infarct. The role of lymphatics in the healing process has not been extensively studied and warrants further investigation in light of these findings. Additional imaging and biological studies are necessary to examine the role of the myocardial lymphatic system in trafficking of MSCs in the heart.

## Methods

### Study groups

The protocol of studies in experimental animals number 10-05-12022 has been approved by the Institutional Animal Care and Use Committee of the UT MD Anderson Cancer Center, Houston, TX. Adult pigs *Sus scrofa* (N = 9) were divided into 4 groups. **Group I** pigs (negative control; n = 3) received injections of nontransduced autologous MSCs. **Group II** pigs (positive control; n = 2) received sr39HSV1tk-Wi-86 positive control cells (4–6 injections of 1×10^8^ in 0.1 ml of PBS per injection into the left ventricular apical septum). **Group III** pigs (test group; n = 3) received autologous sr39HSV1-tk-MSC (3 injections of 1×10^8^ cells in 0.1 ml of PBS per injection into the apical or subcuspidal area of the left ventricular septum) 7 days after infarction. The one pig in **Group IV** (PG-Gd-NIRF813 study group) at day 7 post infarct received polyglutamic-Gd-DTPA near-infrared fluorescence (PG-Gd-NIRF813) contrast agent [51] to map the lymphatic drainage from the injection site in the myocardium. PG-Gd-NIRF813 was injected into the same site (1 mg in 0.5 ml saline) as the sr39HSV1tk-MSCs.

### Isolation, expansion, and characterization of porcine MSCs

All work was performed under approved Institutional Animal Care and Use Committee (IACUC) protocols. Briefly, 10–20 ml of bone marrow was aspirated under local anesthesia from the iliac crest of pigs (weighing about 100 kg each) 30 days before imaging experiments. The aspirates were heparinized and centrifuged at 800× *g* for 10 min at room temperature; the serum was discarded. The cells were diluted 1∶1 with phosphate-buffered saline (PBS), layered over an equal volume of Ficoll (1.077 g/mL, Technoclones), and centrifuged at 800× *g* for 30 min. Mononuclear cells recovered from the buffy coat at the gradient interface were washed twice with PBS (300× *g* for 5 min at room temperature). Cells were seeded at 2×10^5^/cm^2^ in alpha modified Eagle's medium (aMEM) (Gibco) with 20% fetal bovine serum (FBS) (Gibco). The cells were then cultured at 37°C in 5% CO_2_ in air, and nonadherent cells were removed after 5 days. The culture medium was replaced every 3 to 4 days. After about 7 days, when isolated colonies of pig MSCs were apparent, the cells were trypsinized and replated at 8000/cm.^2^ Immunophenotyping of MSCs was performed by using the following human antibodies conjugated with fluorescin isothiocyanate (FITC) or phycoerythrin (PE): CD44, CD90, pig SLA1, CD31, and CD45 (Becton-Dickinson); VEGFR-3/Flt-4 and CCR7 (R&D Systems); unconjugated LYVE-1 rabbit antibody (AngioBio) was recognized by a secondary AlexaFluor-488-conjugated goat-anti-rabbit antibody (Invitrogen). Human-IgG1-FITC, and IgG1-PE were used as isotype controls. In addition, pig MSCs (pre- and post-viral transduction) were subjected to both adipose and bone lineage differentiation assays as described previously [52]. Collectively, these data confirmed the MSC nature of this cell population. Fluorescence-activated cell sorting (FACS) was performed using FACSAria (BD Biosciences) and fluorescence microscopy using BX51 (Olympus).

### Transduction of Porcine MSCs

Stock MND-sr39HSV1tk-LNGFR retroviral producer cells [53] (1×10^7^ PFU/ml) were grown to 70% confluence at 37°C; the producer lines were placed at 32°C, and supernatants were collected the next morning. Porcine MSCs (approximately 3×10^6^/flask) were washed once in PBS, and fresh MND-sr39HSV1tk-LNGFR virus supernatants were added to each T175 flask, together with 8 µg/ml of polybrene. The cultures were rocked slowly overnight, and this process was repeated the next morning with fresh retrovirus-containing supernatant at 32°C. The MSCs were transduced with the retrovirus within the first 4 passages after isolation and initial plating. At 48 hours after transduction, the MSCs were analyzed by flow cytometry for transduction efficiency by using anti-human LNGFR monoclonal antibody (LN-12; Boehringer Mannheim). Transduced MSCs expressing high levels of the LNGFR reporter gene were designated as sr39HSV1tk-MSCs.

### Sr39HSV1tk-Wi-38 control cells

The Wi-38 murine fibroblasts (from ATCC) were stably transduced with the MND-sr39HSV1tk-LNGFR described above by using Fugene (Roche Applied Science), according to the manufacturer's protocol. After antibiotic selection, the remaining cells were sorted via FACs for high expression levels of LNGFR cell surface reporter, and individual clones were isolated. We selected the clone #86 for coexpression of very high levels of both LNGFR and high sr39HSV1-TK enzyme activity, as described previously. This clone, designated sr39HSV1tk-Wi-38, was grown in DMEM 10% FCS.

### *In vitro* [^3^H]FEAU accumulation assay

Assessment of radiotracer uptake and accumulation was performed as described previously [8], [14]. Briefly, the sr39HSV1tk-MSCs cells were plated in duplicate in 150-mm dishes and grown until about 50% confluent. Then, the culture medium was replaced with 14 mL of medium containing radiotracers [^3^H]FEAU 3.7 kBq/mL (Moravek). The cells were incubated in the [^3^H]FEAU-containing medium for 15, 30, 60, and 120 minutes before scraping the monolayers, transferring the content into 15-mL tubes, and centrifuging at 1000×g for 2 minutes. A 100 µL aliquot of supernatant was transferred to a pre-weighed scintillation tube, and the rest was removed by aspiration before snap-freezing the cell pellet on dry ice. The frozen pellets were transferred to pre-weighed scintillation vials and weighed again to calculate the weight of the cell pellet. The frozen pellets were transferred to pre-weighed scintillation vials, weighed, thoroughly dissolved in 0.5 mL of Soluene-350 for 24 hours, and mixed with 5 ml of scintillation cocktail Ultima Gold (Perkin Elmer). Radioactive β-emissions were measured within pre-calibrated energy ranges by using a scintillation counter Packard Tri-Carb 3100TR (Perkin Elmer) to quantify the [^3^H]FEAU and were normalized by the cell pellet weight to calculate the radioactivity concentration (dpm/g). Radioactivity concentration in cell pellets was divided by that of the medium [(dpm/g cells)/(dpm/g medium)] and plotted against time. A linear regression analysis (regression slope) was used to calculate the rate of [^3^H]FEAU accumulation in cells, which is proportional to the level of sr39HSV1-tk gene expression.

### Porcine model of acute myocardial infarction

This model has been described previously [54], [55]. Briefly, a catheter was advanced through the aortic valve into the left ventricular cavity. Aortic and ventricular pressures were recorded. A left ventriculogram was performed in a 30° RAO projection by using a controlled injection of 30 ml of a nonionic, iso-osmolar contrast agent (Visipaque, GE Healthcare) at a constant rate of 10 ml/sec. A 6F coronary guiding catheter was advanced through the aorta and was selectively engaged into the left coronary ostium. Angiograms were performed at the predetermined projections: PA, 30° LAO, and 30° RAO. A floppy 0.014″ coronary guide wire was positioned at the distal third of the left anterior descending artery. An angioplasty balloon approximately the same size as the artery was placed and was inflated to nominal pressure. Contrast was injected to ensure the complete occlusion of the distal bed and the patency of the diagonal branch. The balloon remained inflated for approximately 90 minutes and then was deflated to allow for passive reperfusion. The pig was transferred to the ICU for monitoring.

### NOGA-mediated transendomyocardial injection of sr39HSV1-tk-MSC or sr39HSV1tk-Wi-38 cells

Nonfluoroscopic electromechanical mapping has been described [54]. Briefly, an 8F NOGA Myostar catheter (Cordis) was introduced through the left femoral sheath 7 days after MI, and left ventricular electromechanical mapping was performed. Mapping points were captured by measuring the unipolar voltage of the endocardium to construct a 3D representation of the left ventricle. During the procedure, brief (<3 s) nonsustained ventricular tachycardia was observed in some pigs and was attributed to irritation of the left ventricular endocardium from catheter navigation. The NOGA injection catheter was prepared by adjusting the needle extension at 0° and 90° flex. The injection catheter tip was placed across the aortic valve and into the apical septum; each injection site was carefully evaluated before cell injection.

### PET/CT imaging with [^18^F]FEAU

The synthesis of [^18^F]FEAU has been described [56], [57]. [^18^F]-FEAU was administered intravenously at a dose of 11.99±1.79 mCi/animal. Repeat PET/CT imaging studies were performed on a GE Discovery ST-8 PET-CT scanner (GE Healthcare). A conventional, noncontrast CT scan (300 mA; 120 kVp; 0.5 sec X-ray tube rotation, table speed, 13.5 mm/rotation) was acquired. Then, dynamic PET images of the thorax were acquired in 2-dimensional mode following the administration of 16 mCi of [^18^F]FEAU for a period of 0–40 min. This was then followed by two whole body PET images acquired at 40 and 60 min post [^18^F]FEAU administration using 4 bed positions, 5 min each. PET images were reconstructed by using VUE Point reconstruction software (GE Healthcare), which uses an ordered subset expectation maximization (OSEM) algorithm. Emission data were corrected for attenuation scatter (with the use of CT), random events, and dead-time losses by using manufacturer's software. The CT and PET data were transferred to a dedicated imaging analysis workstation (Hermes Browser, Version 3.0, Hermes Medical Solutions). The PET/CT images were re-oriented to match the cardiac apex and to align the cardiac axis using the rib cage (CT images) as an external anatomical reference using the Hybrid Viewer software module (Hermes). Images were cross-normalized by using the Volume Display Program, Version 1.2 (Hermes) to accurately compare images obtained at different days of the study. Circular regions of interest (ROI) were placed in the areas with the highest radioactivity and in the muscle tissue with a low level of activity. Maximum SUV was calculated from each ROI by using the following formula: SUV = measured activity concentration (Bq/g)×body weight (g)/injected activity (Bq). For the assessment of radioactivity accumulation in the lungs, the lung area in each transaxial plane on the PET image was outlined manually on the basis of corresponding MRI and/or CT images.

### Echocardiography

Myocardial contractile function was assessed by using echocardiography on the day of [^18^F]FEAU PET/CT, as previously described [58], [59]. The heart was imaged in the 2D mode in the short-axis view of the left ventricle at the level of the papillary muscle. The left ventricular ejection fraction was obtained according to the American Society of Echocardiography leading-edge method from at least 3 consecutive cardiac cycles [60]. Similar studies with [^18^F]FDG and ultrasound in a pig model of MI have been reported previously [61].

### *In vivo* MR imaging

MRI lymphography with polyglutamic-Gd-DTPA near-infrared fluorescence (PG-Gd-NIRF813) contrast agent was used to study the role of lymphatic drainage in the trafficking of sr39HSV1tk-MSCs from the injection site in the myocardium and persistence of sr39HSV1tk-MSCs in the heart. The synthesis of PG-Gd-NIRF813 has been described [51], [62]. After MI, PG-Gd-NIRF813 (250 mg in 4 mL PBS) was injected into the same sites as those described above for MSC injection. PG-Gd-NIRF813 was injected into the right shoulder for reference. MRI was performed at 2 hours and repeated at 24 hours after PG-Gd-NIRF813 injection by using an 8-channel 1.5T whole-body research MR scanner (Signa HDx; General Electric Healthcare) with a phased-array coil and both cardiac and respiratory gating. Fat-suppressed, T1-weighted, Dixon-based in- and out-of-phase FIESTA cine and double IR imaging were performed. Fields of view included short-axial and 4-chamber views of the heart, as well as axial and coronal views of the thorax.

### *Ex vivo* MRI

The heart was resected immediately after *in vivo* MR imaging, and *ex vivo* MR imaging was performed on a 4.7 Tesla GE/Bruker Biospec scanner. Axial and coronal images were obtained by using a 100-mT/m, 26-cm-inner-diameter, actively shielded gradient coil system (285.71 T/m-s slew rate) and a 19.7-cm-inner-diameter, volume radiofrequency coil. T1-weighted MR images (TE = 10.5 ms, TR = 800 ms) were acquired with a 14.5×10.87 cm field-of-view coronal and a 12×9 cm field-of-view axial; 2-mm section thickness, 2-mm gap, and a 256×192 matrix.

### *Ex vivo* fluorescence imaging

The resected heart and lymph nodes were imaged by using an IVIS-200 imaging system (Xenogen Corp.) with ICG filter sets (excitation/emission, 710–760/810–875 nm). The camera setting included medium binning (8*) with an F stop of 1. The CDD resolution was 2048×2048 pixels; the exposure time was 3 seconds, and the field of view was 13×13 cm.

### Immunohistochemistry

Control pigs were euthanized 2 days after MSC injections, and experimental pigs were euthanized at 35 days (n = 2) and 150 days (n = 1) after injection. Tissue samples from the regions with high levels of high [^18^F]FEAU accumulation on PET/CT images, including the heart and the paraaortic and cervical lymph nodes, were fixed in 2% paraformaldehyde and embedded in paraffin. Serial paraffin sections were deparaffinized, rehydrated, and pressure-cooked for 4 min in citrate buffer (10 mM, pH 6.0) for antigen retrieval. The sections were incubated in 1% H_2_O_2_/methanol and then in 4% goat serum (Abnova). Primary monoclonal antibodies against HSV1-TK protein (clone 4C8 at 1∶50 dilution, from Dr. William Summers, Yale University), CD71 (1∶50, Santa Cruz), CD90 (1∶50, Santa Cruz), vimentin (1∶50, Santa Cruz), α-smooth muscle actin (αSMA; Santa Cruz), and LYVE-1 (AngioBio) were used. Secondary antibodies conjugated to horseradish peroxidase or alkaline phosphatase (Vector, CA) were used to detect primary antibody reactive tissues and were stained with 3′3′-diaminobezidine (DAB) and 5-bromo-4-chloro-3-indolyl phosphate/nitroblue tetrazolium (BCIP/NBT), respectively. Adjacent tissue sections were stained with hematoxylin and eosin.

## Supporting Information

Figure S1 **The fate of intramyocardially injected transduced sr39HSV1tk-MSCs monitored over 35 days with [^18^F]FEAU PET/CT in pig #8s210.** (**A**) The level of LNGFR co-expression with sr39HSV1-TK in transduced (green) and nontransduced (purple) sr39HSV1tk-MSCs. (**B**) [^3^H]FEAU accumulation over time in sr39HSV1tk-MSCs (influx rate Ki = 0.092±0.006 ml/g/min) reflects a moderate level of *sr39HSV1-tk* reporter gene expression in this particular cell population. (**C**) NOGA maps indicating sites of sr39HSV1-tk-MSC injection into the myocardium. (**D**) Dynamics of regional [^18^F]FEAU accumulation (SUV) in the site of stem cell injection (blue diamonds), the interventricular septum (blue circles), the paraaortic lymph node(s) (green upward triangles), the proximal left anterior descending coronary artery region (green downward triangles), and the infarct area in the anterior left ventricular wall (red squares). (**E**) Axial PET/CT images of the heart obtained 1 hour after [^18^F]FEAU administration at baseline and at different time points after intramyocardial injection of sr39HSV1tk-MSCs demonstrating the spatial and temporal dynamics of the sr39HSV1tk-MSCs distribution.
(TIF)

Figure S2 **The fate of intramyocardially injected transduced sr39HSV1-tk-MSCs monitored over 35 days with [^18^F]FEAU PET/CT in pig #8s211.** (**A**) The level of LNGFR co-expression with HSV1-TK in transduced (green) and nontransduced (purple) sr39HSV1tk-MSCs. (**B**) [^3^H]FEAU accumulation over time in sr39HSV1-tk-MSCs (influx rate Ki = 0.151±0.005 ml/g/min) reflects a moderate level of *sr39HSV1-tk* reporter gene expression in this particular cell population. (**C**) NOGA maps indicating sites of sr39HSV1-tk-MSC injection into the myocardium. (**D**) Dynamics of regional [^18^F]FEAU accumulation (SUV) in the site of stem cell injection (blue diamonds), the interventricular septum (blue circles), the paraaortic lymph node(s) (green upward triangles), the proximal left anterior descending artery region (green downward triangles),and the infarct area in the anterior left ventricular wall (red squares). (**E**) Axial PET/CT images of the heart obtained 1 hour after [^18^F]FEAU administration at baseline and at different time points after intramyocardial injection of sr39HSV1tk-MSCs demonstrating the spatial and temporal dynamics of the sr39HSV1tk-MSCs distribution.
(TIF)

Figure S3 **[^18^F]FEAU PET/CT images of cervical lymph nodes in a pig after intramyocardial injection of sr39HSV1tk-MSCs.** (**A**) Coronal, sagittal, and axial PET/CT images of cervical region and (**B**) [^18^F]FEAU accumulation in cervical lymph nodes at different time points after intramyocardial injection of sr39HSV1tk-MSCs: points – average SUV for all positive nodes; bars – standard deviation. (**C**) The presence of Sr39HSV1-TK+ cells in cervical lymph nodes was confirmed by IHC.
(TIF)

Figure S4 **Coexpression of sr39HSV1-TK and αSMA in myocardial tissue sections obtained from a pig at 35 days post intramyocardial injection of sr39HSV1tk-MSCs.** Prominent co-localized expression of sr39HSV1-TK (**A,C,E**) and α-SMA (**B,D,F**) was observed in the periinfarct areas of myocardium.
(TIF)

Figure S5 ***Ex vivo*** **T1-weighted MR lymphography of the excised porcine heart obtained at 28 hours after intramyocardial injection of PG-Gd-NIRF contrast.** (**A**) Serial transaxial T1-weighted contrast-enhanced MR images the same heart shown in Fig. 6F demonstrating the localization and extent of myocardial infarct at day 9 post infarct and pathways of lymphatic outflow (white contrast signals). (**B**) NOGA maps of contrast agent injection sites.
(TIF)

Figure S6 ***Ex vivo*** **T1-weighted MR lymphography of the excised heart obtained at 28 hours after intramyocardial injection of PG-Gd-NIRF contrast.** (**A**) Serial sagittal T1-weighted contrast-enhanced MR images of the same heart shown in Fig. 6E demonstrating the localization and extent of myocardial infarct at day 9 post infarct and pathways of lymphatic outflow (white contrast signals). (**B**) NOGA maps of contrast agent injection sites.
(TIF)
