# Supplementary figures and images for: Imaging Long-Term Fate of Intramyocardially Implanted Mesenchymal Stem Cells in a Porcine Myocardial Infarction Model
Source: PLoS One. 2011 Sep 1;6(9):e22949. doi: 10.1371/journal.pone.0022949 (PMC3164664; doi:10.1371/journal.pone.0022949)

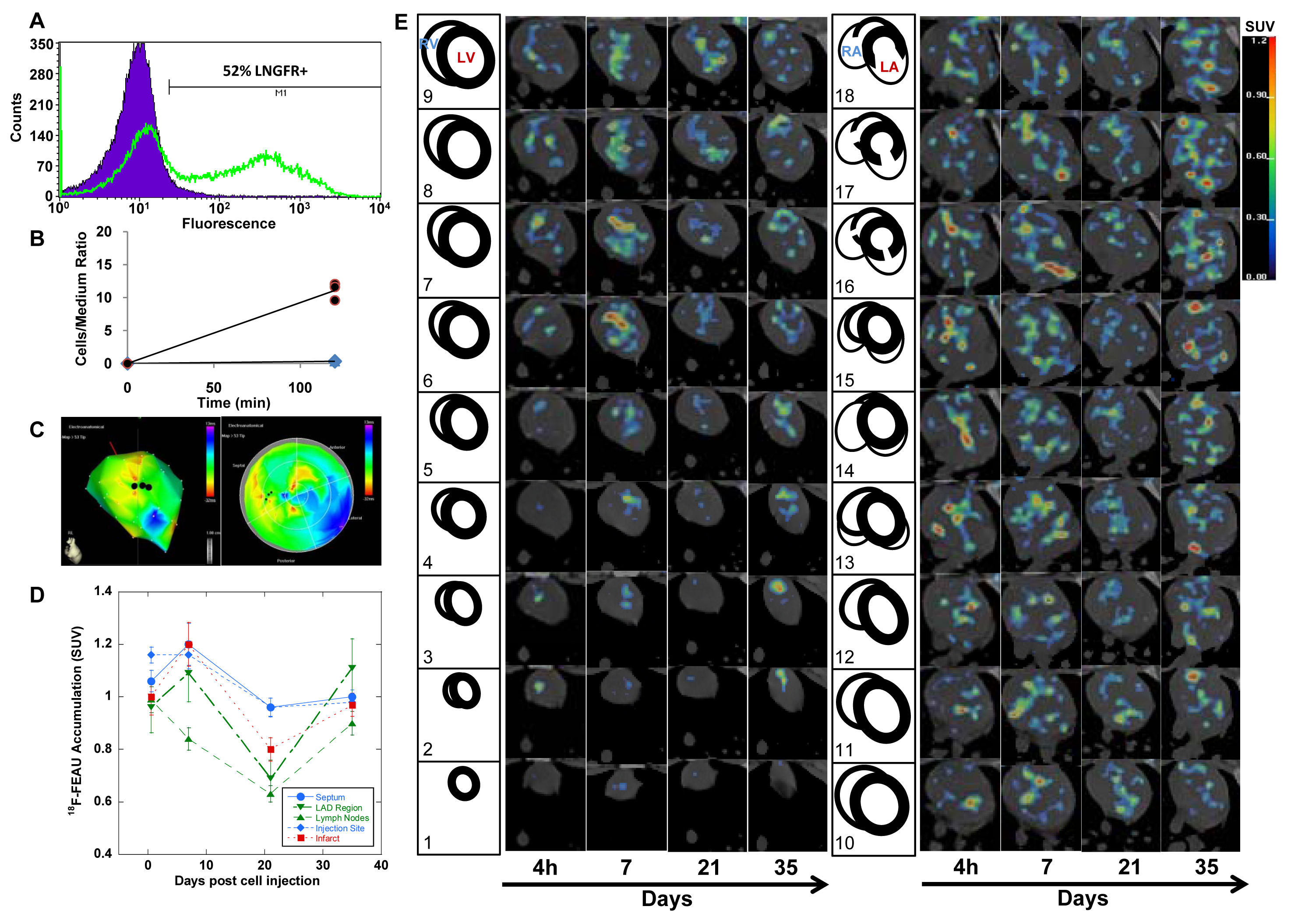

Supplement: Figure S1 — The fate of intramyocardially injected transduced sr39HSV1tk-MSCs monitored over 35 days with [18F]FEAU PET/CT in pig #8s210. (A) The level of LNGFR co-expression with sr39HSV1-TK in transduced (green) and nontransduced (purple) sr39HSV1tk-MSCs. (B) [3H]FEAU accumulation over time in sr39HSV1tk-MSCs (influx rate Ki = 0.092±0.006 ml/g/min) reflects a moderate level of sr39HSV1-tk reporter gene expression in this particular cell population. (C) NOGA maps indicating sites of sr39HSV1-tk-MSC injection into the myocardium. (D) Dynamics of regional [18F]FEAU accumulation (SUV) in the site of stem cell injection (blue diamonds), the interventricular septum (blue circles), the paraaortic lymph node(s) (green upward triangles), the proximal left anterior descending coronary artery region (green downward triangles), and the infarct area in the anterior left ventricular wall (red squares). (E) Axial PET/CT images of the heart obtained 1 hour after [18F]FEAU administration at baseline and at different time points after intramyocardial injection of sr39HSV1tk-MSCs demonstrating the spatial and temporal dynamics of the sr39HSV1tk-MSCs distribution. (TIF) [file pone.0022949.s001.tif]

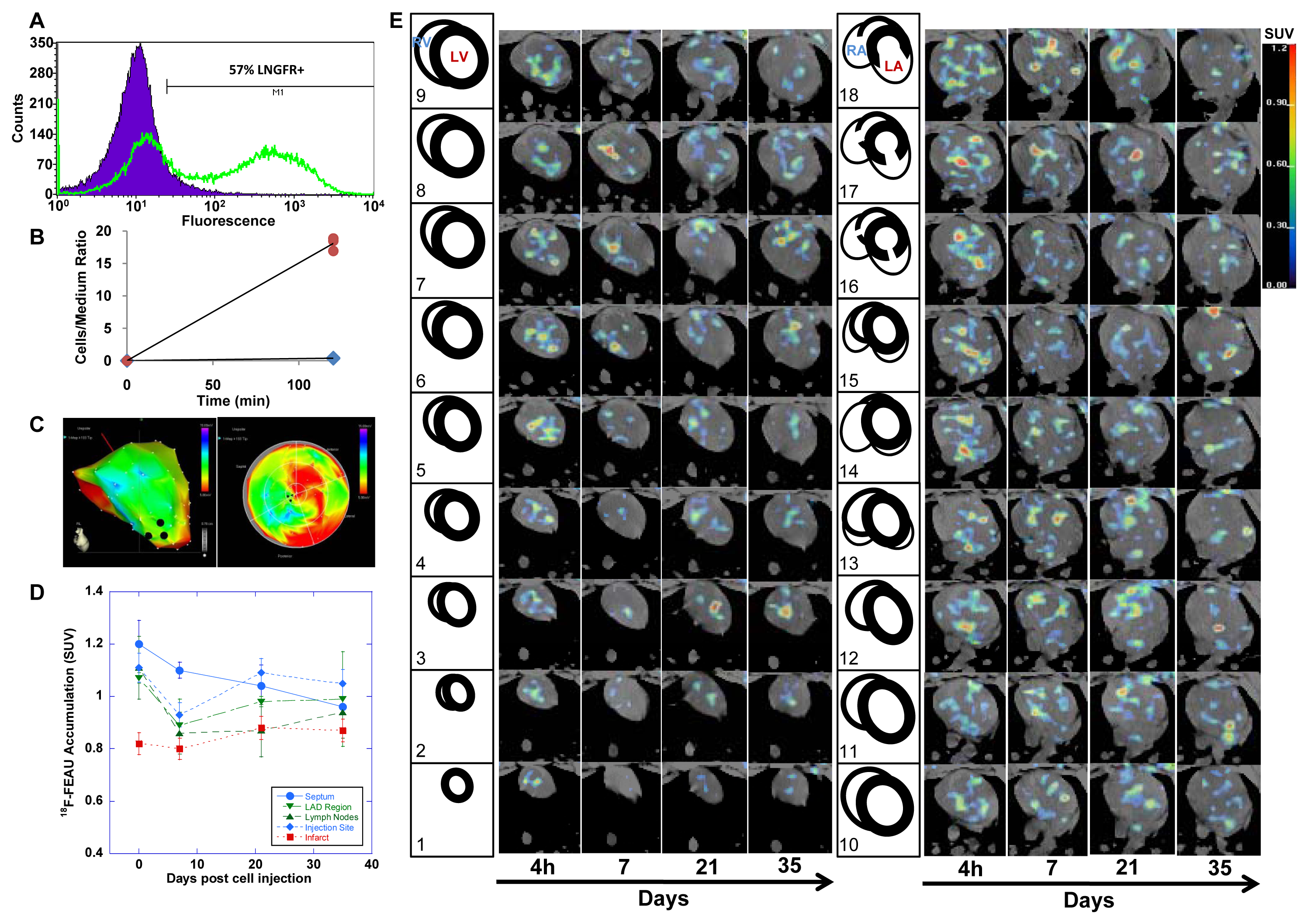

Supplement: Figure S2 — The fate of intramyocardially injected transduced sr39HSV1-tk-MSCs monitored over 35 days with [18F]FEAU PET/CT in pig #8s211. (A) The level of LNGFR co-expression with HSV1-TK in transduced (green) and nontransduced (purple) sr39HSV1tk-MSCs. (B) [3H]FEAU accumulation over time in sr39HSV1-tk-MSCs (influx rate Ki = 0.151±0.005 ml/g/min) reflects a moderate level of sr39HSV1-tk reporter gene expression in this particular cell population. (C) NOGA maps indicating sites of sr39HSV1-tk-MSC injection into the myocardium. (D) Dynamics of regional [18F]FEAU accumulation (SUV) in the site of stem cell injection (blue diamonds), the interventricular septum (blue circles), the paraaortic lymph node(s) (green upward triangles), the proximal left anterior descending artery region (green downward triangles),and the infarct area in the anterior left ventricular wall (red squares). (E) Axial PET/CT images of the heart obtained 1 hour after [18F]FEAU administration at baseline and at different time points after intramyocardial injection of sr39HSV1tk-MSCs demonstrating the spatial and temporal dynamics of the sr39HSV1tk-MSCs distribution. (TIF) [file pone.0022949.s002.tif]

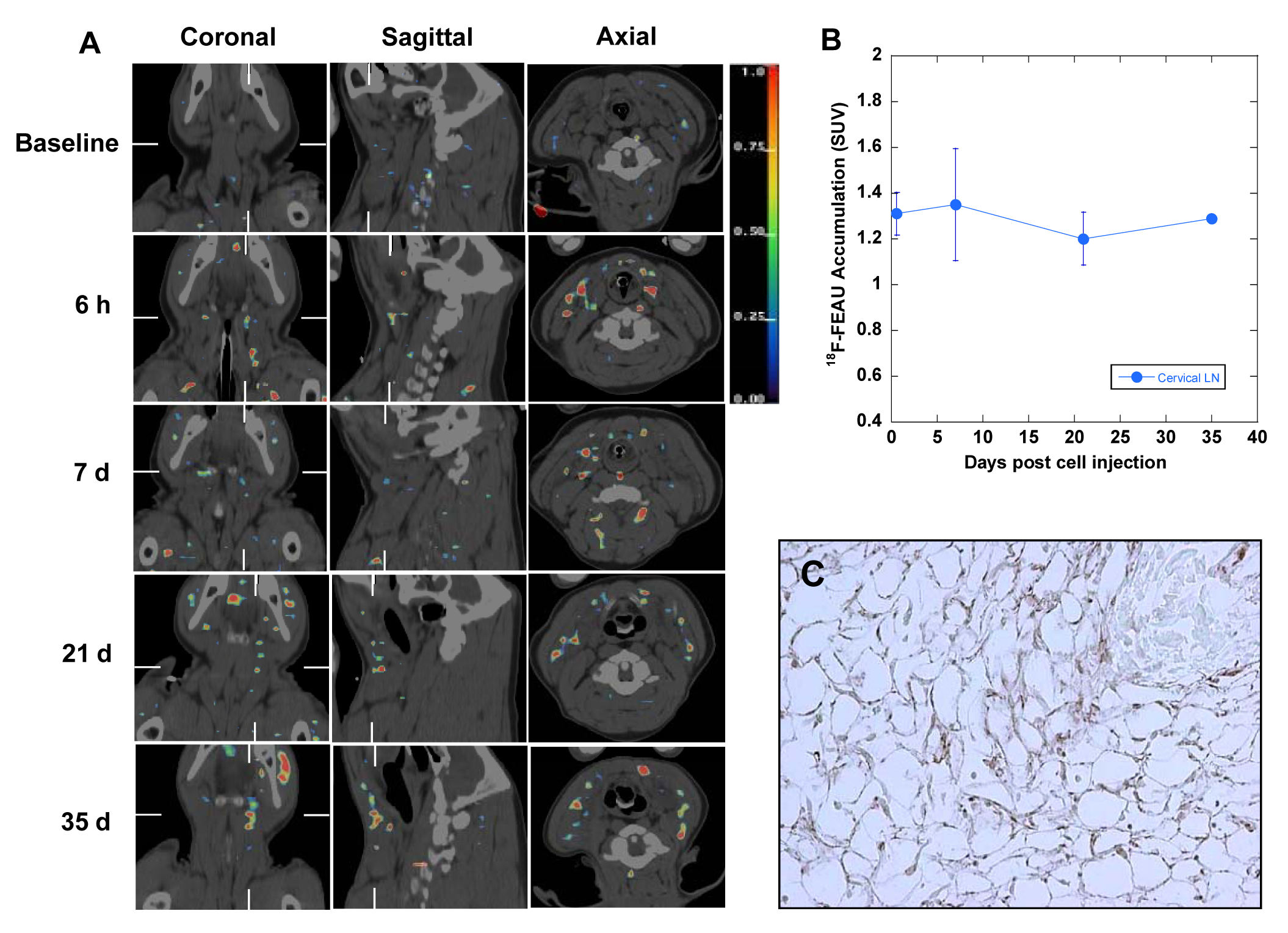

Supplement: Figure S3 — [18F]FEAU PET/CT images of cervical lymph nodes in a pig after intramyocardial injection of sr39HSV1tk-MSCs. (A) Coronal, sagittal, and axial PET/CT images of cervical region and (B) [18F]FEAU accumulation in cervical lymph nodes at different time points after intramyocardial injection of sr39HSV1tk-MSCs: points – average SUV for all positive nodes; bars – standard deviation. (C) The presence of Sr39HSV1-TK+ cells in cervical lymph nodes was confirmed by IHC. (TIF) [file pone.0022949.s003.tif]

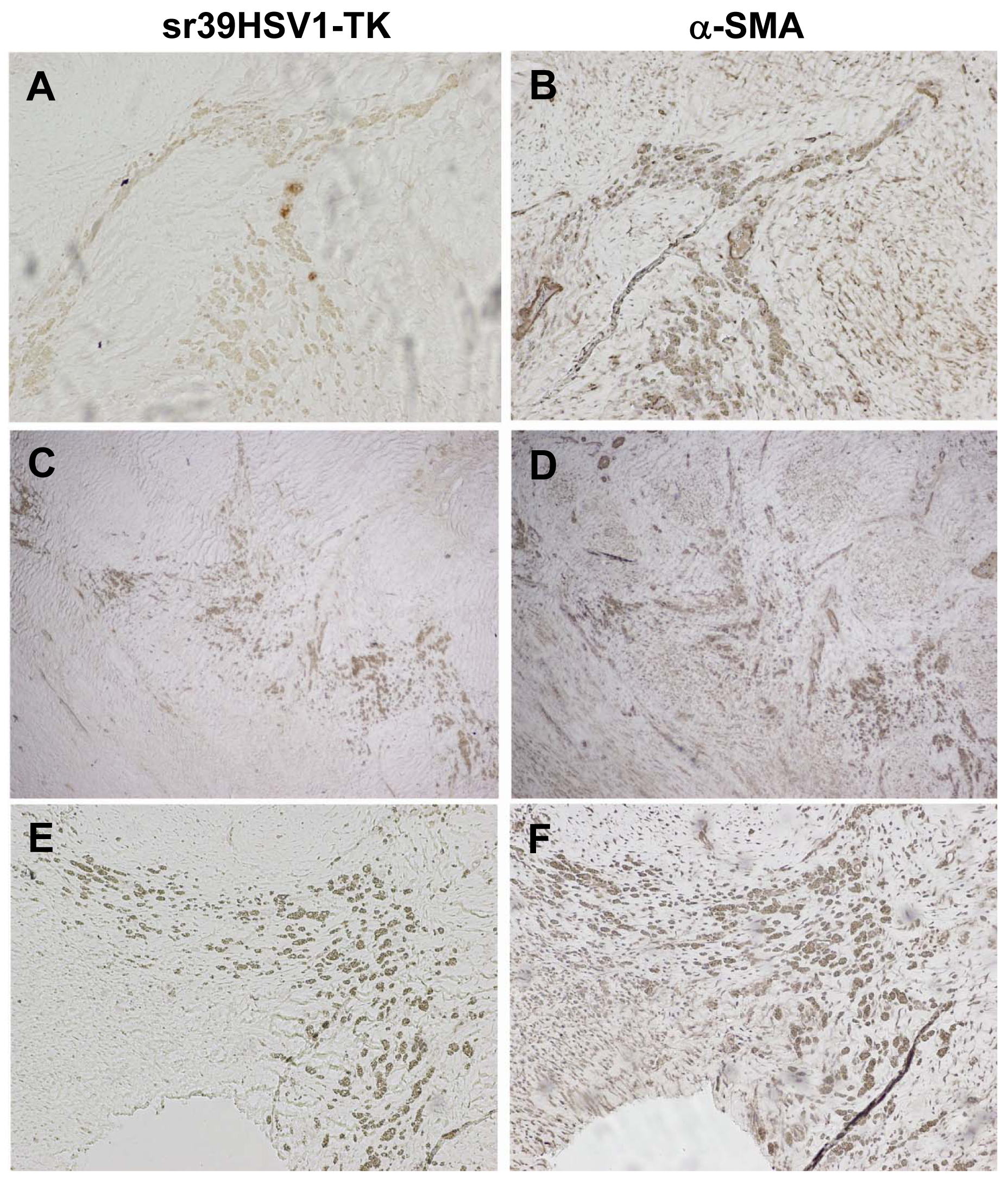

Supplement: Figure S4 — Coexpression of sr39HSV1-TK and αSMA in myocardial tissue sections obtained from a pig at 35 days post intramyocardial injection of sr39HSV1tk-MSCs. Prominent co-localized expression of sr39HSV1-TK (A,C,E) and α-SMA (B,D,F) was observed in the periinfarct areas of myocardium. (TIF) [file pone.0022949.s004.tif]

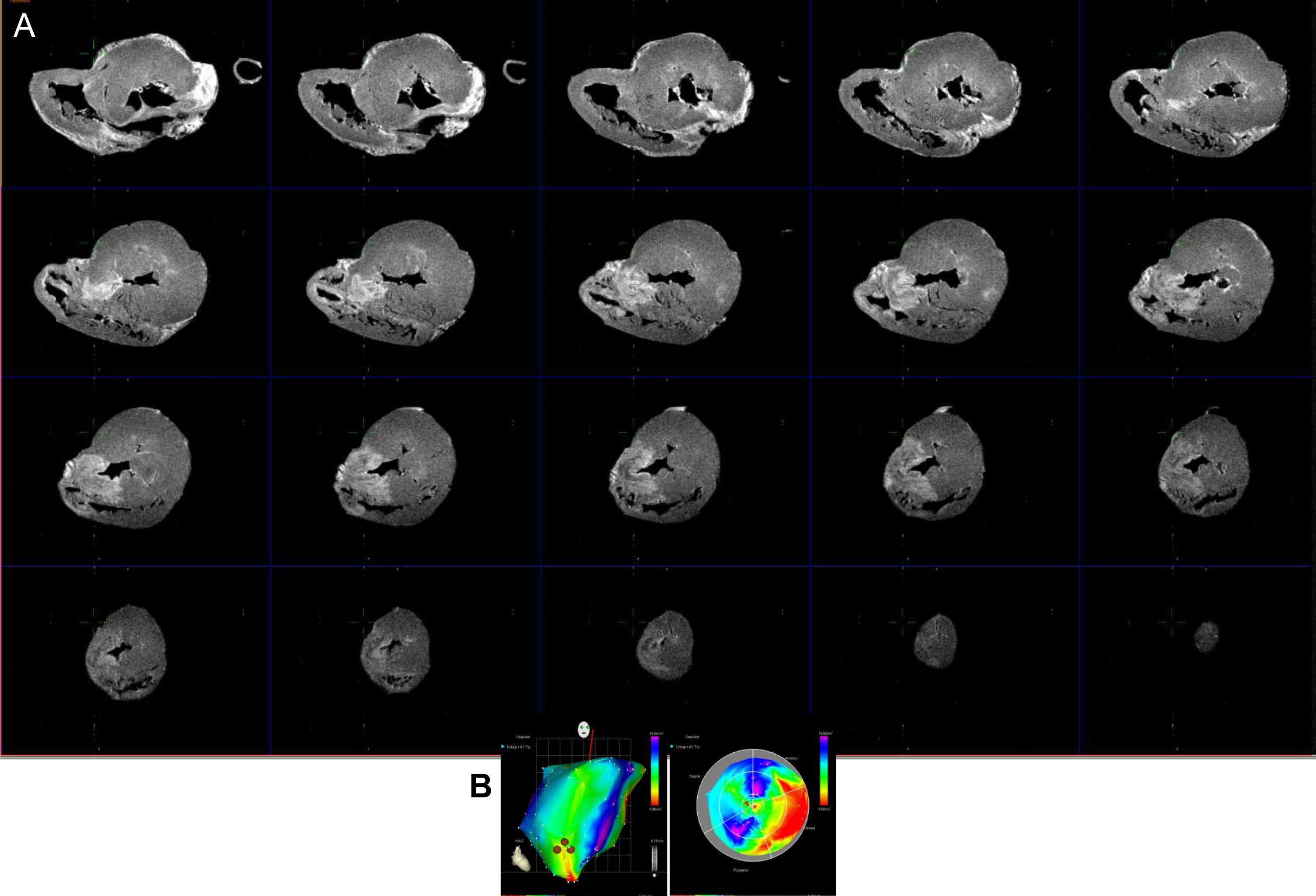

Supplement: Figure S5 — Ex vivo T1-weighted MR lymphography of the excised porcine heart obtained at 28 hours after intramyocardial injection of PG-Gd-NIRF contrast. (A) Serial transaxial T1-weighted contrast-enhanced MR images the same heart shown in Fig. 6F demonstrating the localization and extent of myocardial infarct at day 9 post infarct and pathways of lymphatic outflow (white contrast signals). (B) NOGA maps of contrast agent injection sites. (TIF) [file pone.0022949.s005.tif]

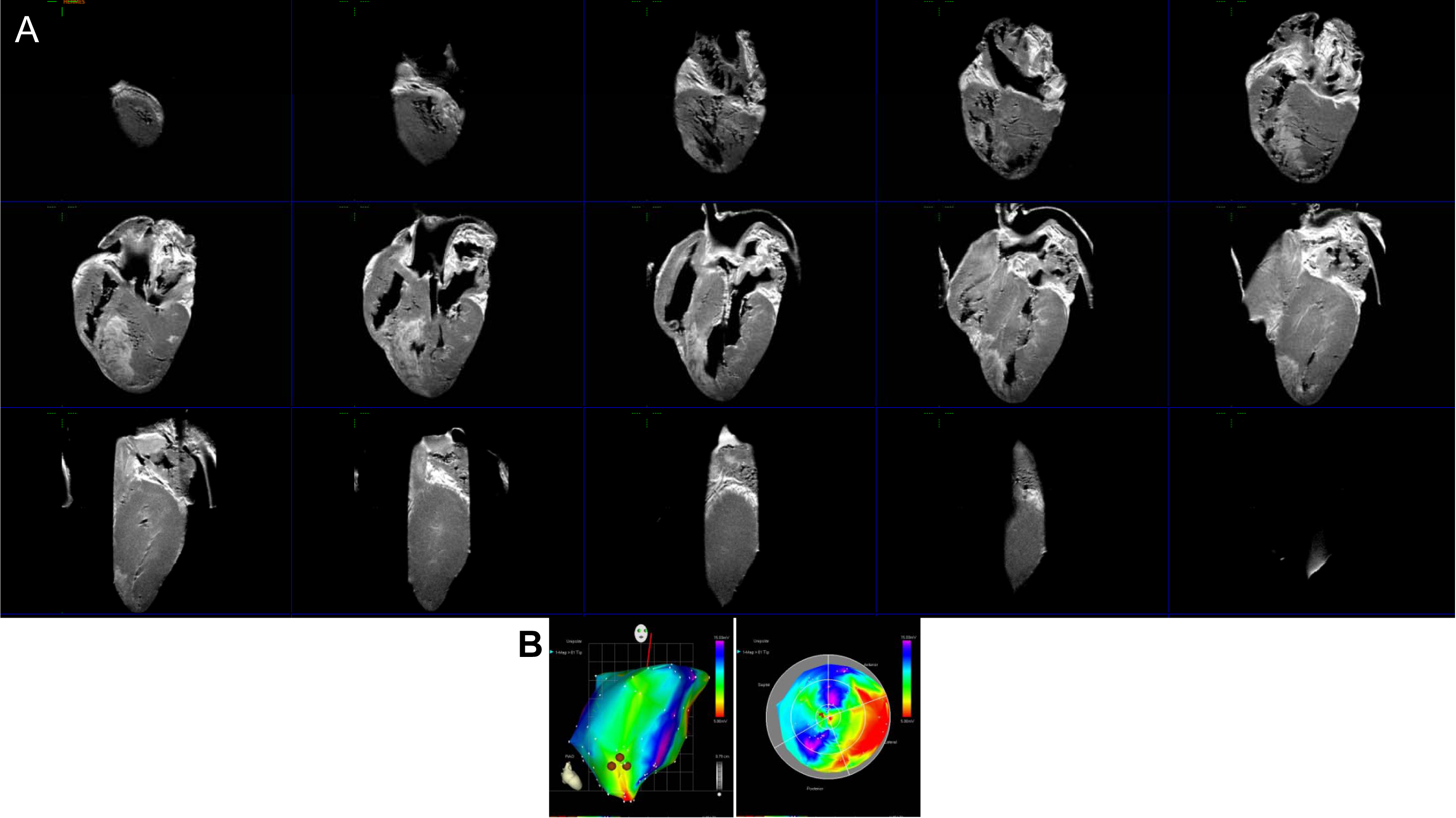

Supplement: Figure S6 — Ex vivo T1-weighted MR lymphography of the excised heart obtained at 28 hours after intramyocardial injection of PG-Gd-NIRF contrast. (A) Serial sagittal T1-weighted contrast-enhanced MR images of the same heart shown in Fig. 6E demonstrating the localization and extent of myocardial infarct at day 9 post infarct and pathways of lymphatic outflow (white contrast signals). (B) NOGA maps of contrast agent injection sites. (TIF) [file pone.0022949.s006.tif]
